# Karyotypes and Sex Chromosomes in Two Australian Native Freshwater Fishes, Golden Perch (*Macquaria ambigua*) and Murray Cod (*Maccullochella peelii*) (Percichthyidae)

**DOI:** 10.3390/ijms20174244

**Published:** 2019-08-30

**Authors:** Foyez Shams, Fiona Dyer, Ross Thompson, Richard P. Duncan, Jason D. Thiem, Zuzana Majtánová, Tariq Ezaz

**Affiliations:** 1 Institute for Applied Ecology, Faculty of Science and Technology, University of Canberra, Canberra, Australian Capital Territory 2617, Australia; 2 Department of Primary Industries, Narrandera Fisheries Centre, Narrandera, New South Wales 2700, Australia; 3 Institute of Animal Physiology and Genetics, CAS, p.r.i., 277 21 Liběchov, Czech Republic

**Keywords:** sex determination, florescence in situ hybridisation (FISH), comparative genomic hybridisation (CGH), karyogram, DNA methylation

## Abstract

Karyotypic data from Australian native freshwater fishes are scarce, having been described from relatively few species. Golden perch (*Macquaria ambigua*) and Murray cod (*Maccullochella peelii*) are two large-bodied freshwater fish species native to Australia with significant indigenous, cultural, recreational and commercial value. The arid landscape over much of these fishes’ range, coupled with the boom and bust hydrology of their habitat, means that these species have potential to provide useful evolutionary insights, such as karyotypes and sex chromosome evolution in vertebrates. Here we applied standard and molecular cytogenetic techniques to characterise karyotypes for golden perch and Murray cod. Both species have a diploid chromosome number 2*n* = 48 and a male heterogametic sex chromosome system (XX/XY). While the karyotype of golden perch is composed exclusively of acrocentric chromosomes, the karyotype of Murray cod consists of two submetacentric and 46 subtelocentric/acrocentric chromosomes. We have identified variable accumulation of repetitive sequences (AAT)_10_ and (CGG)_10_ along with diverse methylation patterns, especially on the sex chromosomes in both species. Our study provides a baseline for future cytogenetic analyses of other Australian freshwater fishes, especially species from the family Percichthyidae, to better understand their genome and sex chromosome evolution.

## 1. Introduction

Morphological characteristics of chromosomes, like the size and number (including ploidy) may vary within taxonomic groups such as families, genera and even species [1]. Moreover, functional differences (e.g., accumulation of repetitive sequences, location of a certain gene or DNA methylation) often occur among species as well as among populations or among individuals of the same species [2,3,4]. Consequently, cytogenetic studies provide a useful tool for establishing phylogenetic and evolutionary relationships among species. Karyotype analysis can also facilitate the discovery of heteromorphic or heterochromatic sex chromosomes.

Identification of sex chromosomes is important in dioecious species as it provides information on the evolution of sex determination and insights into the effect of environment in driving sex ratios of populations through upregulation and downregulation of genes in gonadal development [5,6]. The importance of understanding the sex chromosome systems in fish is heightened by the existence of diverse genetic and epigenetic features such as genetic sex determination (GSD), environmental sex determination (ESD), mixed GSD–ESD and rapid sex reversal within this large group of animals [7].

Fish comprise more than fifty percent of extant vertebrate species [8], with around 32,500 species, including more than 15,000 freshwater species [9]. Freshwaters are thus hyperdiverse, considering this habitat constitutes less than 0.3% [10] of available global water. Freshwater fish are thus an important component of global biodiversity and evolutionary studies.

**Figure 1:**
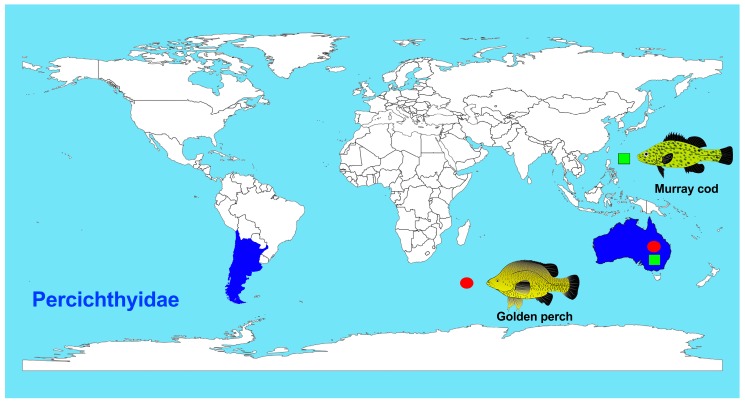
Distribution of nine genera of Percichthyidae covering Australia, Chile and Argentina.

The family Percichthyidae (Order: Centrarchiformes) or temperate perches, consists of 22 freshwater and estuarine species under nine genera, distributed in Australia and South America (Figure 1) [11]. The species investigated here are golden perch (*Macquaria ambigua*) and Murray cod (*Maccullochella peelii*), two large-bodied freshwater fish species native to the Murray Darling Basin (MDB) in south-eastern Australia [12,13]. Golden perch also occurs in central Australia in the Lake Eyre Basin and in the central, coastal Queensland Fitzroy Basin, although these populations are considered to be separate cryptic species [14]. Both golden perch and Murray cod have significant indigenous, cultural, recreational and commercial value. Moreover, both species have been bred in hatcheries and extensively stocked in catchments across the MDB for more than 30 years.

Sex chromosomes in fish have been studied since the early twentieth century, when the presence of heterogametic sex chromosome systems (either XY or WZ) was described [15,16,17]. Studies in fishes have identified diverse sex chromosomal systems (including XX/XY, XX/X0, ZZ/ZW, ZZ/Z0, XX/XY1Y2, X1X1X2X2/X1X2Y, ZZ/ZW1W2, Z1Z1Z2Z2/Z1Z2W), with the XX/XY (male heterogametic) and ZZ/ZW (female heterogametic) systems being predominant [18,19]. The ancestral fish sex chromosome (sex-determining locus) has gone through a complete shift of chromosome pairs because of frequent chromosome rearrangements such as fusion and translocation or transposition [20,21]. Such rearrangement events across the genome, including the sex chromosomes, make teleost fish potentially informative for studies of karyotype and sex chromosome evolution. To date, there have been no published studies on the mode of sex determination in Australian freshwater fish.

**Figure 2:**
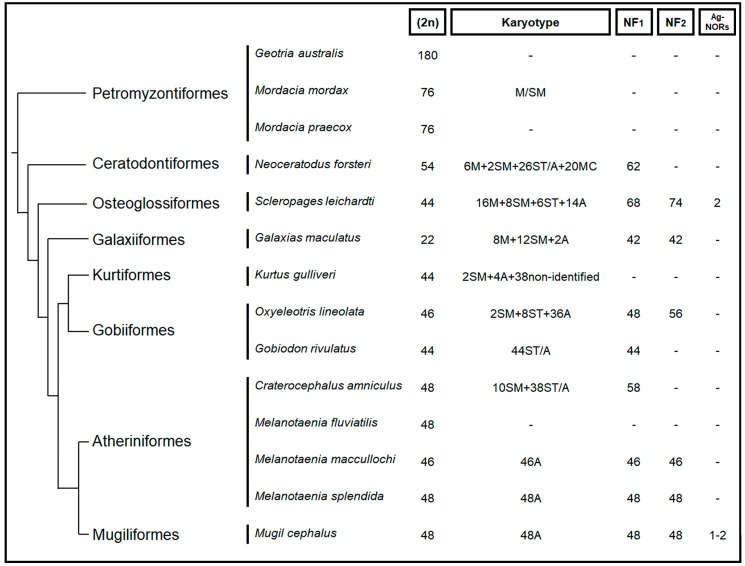
Summary of cytogenetic studies of Australian fish showing the nature of chromosomes from Arai [24], Ezaz, et al. [22] and Majtánová, et al. [23]. Phylogeny adopted from Betancur-R, et al. [25]; M = Metacentric; SM = Submetacentric; ST = Sub-telocentric; A = Acrocentric; MC = Micro Chromosome; NF_1_ = Fundamental arm number, when M and SM are counted as two-armed; NF_2_ = Fundamental arm number, when M, SM and ST are counted as two-armed; Ag-NORs = Number of transcriptionally active nucleolus organiser regions containing chromosomes.

The karyotype data for Australian freshwater fish fauna are scarce (Figure 2) [22,23]. Prior to the current study, there have been no published accounts of karyotype data for any fish from the family Percichthyidae. Molecular characterisation of karyotypes is an important first step towards understanding genome evolution and organisation. In particular, mapping repetitive sequences such as microsatellite associate repeats as well as telomeres can provide an overall landscape of repetitive sequences, as well their role in karyotype evolution, including sex chromosomes. Mapping telomere repeats also provides information about possible chromosome rearrangement events through identification of interstitial telomeric sequences (ITSs).

Here, we describe the karyotypes of golden perch and Murray cod and identify their sex chromosome systems. The karyotype data provide an important first step toward better understanding the evolution of Percichthyids fishes specifically, and Australian freshwater fish more broadly. Analyses of sex chromosome systems in these species will provide insight into sex chromosome evolution in fish and provide the basis for developing tools for conservation and management, such as the identification of sex-linked markers and methods for nondestructive sexing. Our study also provides baseline information to understand the evolutionary trends of karyomorphology in this group.

## 2. Results

### 2.1. Karyotype and C-Banding of Golden Perch and Murray Cod

**Figure 3:**
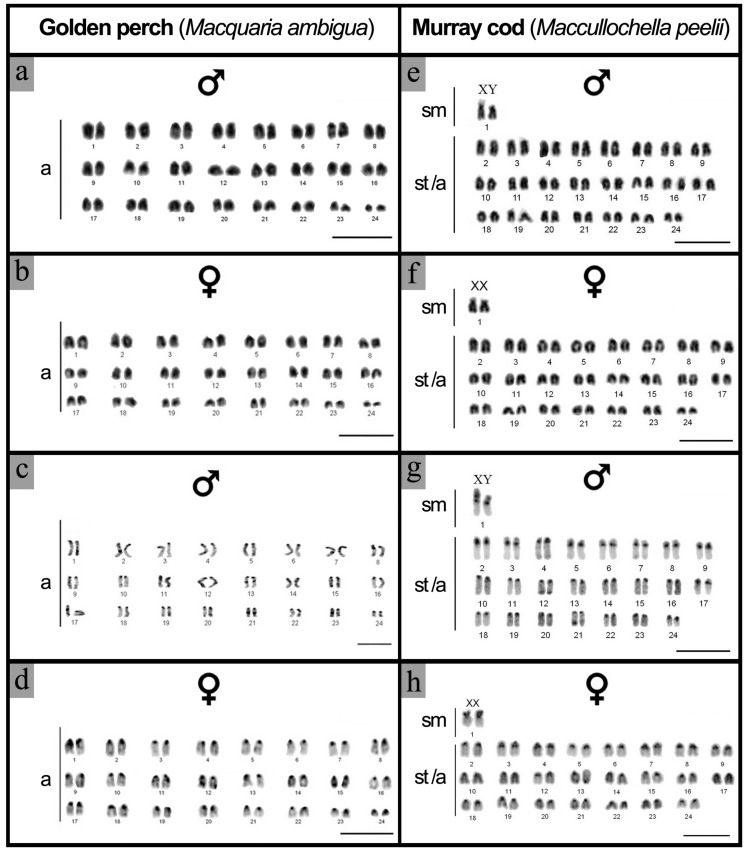
DAPI (4′,6-diamidino-2-phenylindole)-stained and C-banded metaphase karyotypes of golden perch and Murray cod; (**a**) golden perch male karyotype; (**b**) golden perch female karyotype; (**c**) C-banded karyotype of golden perch male; (**d**) C-banded karyotype of golden perch female; (**e**) Murray cod male karyotype; (**f**) Murray cod female karyotype; (**g**) C-banded karyotype of Murray cod male; (**h**) C-banded karyotype of Murray cod female. DAPI-stained metaphases were inverted. Scale bar 5 µm.

The DAPI (4′,6-diamidino-2-phenylindole)-stained metaphase karyotypes were examined in five individuals from each sex of golden perch and three females and five males from Murray cod. For each individual, a minimum of 60 at metaphase cells were examined (Table 1). For both species, the diploid chromosome complement is 2*n* = 48. The golden perch karyotype consisted of 24 pairs of acrocentric chromosomes (Figure 3a,b), while the Murray cod karyotype consisted of one pair of submetacentric and 23 pairs of subtelocentric/acrocentric chromosomes (Figure 3e,f). Comparison of the karyotypes from males and females did not reveal any morphologically differentiated sex chromosomes in golden perch (Figure 3a,b). However, heteromorphism was observed between the largest chromosome pair (submetacentric) of Murray cod. In Murray cod, one chromosome from the largest (submetacentric) pair was shorter in the male karyotype, while in females, the largest chromosome pair was equal in size, suggesting a male heterogametic sex determining system (XX/XY) in this species (Figure 3e,f). This heteromorphism was present in all five males examined and was not observed in any of the three females analysed (Table 1).

C-positive heterochromatin was found in all chromosomes, with C-positive bands near the centromeric regions (Figure 3c,d,g,h) in all animals examined (Table 1). No distinct staining of heterochromatin on any specific chromosome was observed in either males or females of golden perch (Figure 3c,d). On the other hand, a strong C-positive band was observed in the centromeres of all the subtelocentric/acrocentric chromosomes of Murray cod (Figure 3g,h), with a comparatively larger C-positive heterochromatic block on the p arms of the putative sex chromosome pair (heteromorphic pair) (Figure 3g,h).

### 2.2. Intraspecies Comparative Genomic Hybridisation (CGH)

**Figure 4:**
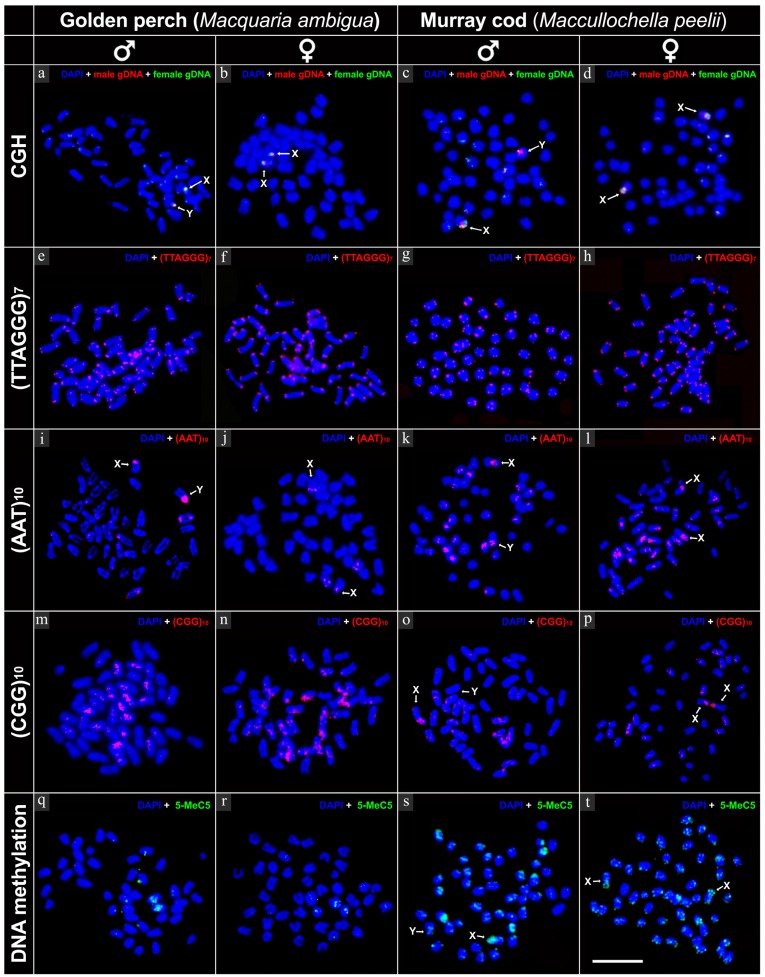
Fluorescence in situ hybridisation signals of genomic DNA (CGH, **a**–**d**), telomeric probe (**e**–**h**), microsatellite motifs (**i**–**p**) and DNA methylation patterns in golden perch and Murray cod (**q**–**t**). CGH (**a**–**d**): hybridisation signals for fluorescently labelled genomic DNA of golden perch and Murray cod, showing hybridisation signals on X and Y sex chromosome; TTAGGG_7_ (**e**–**h**): showing terminal hybridisation signals in all chromosomes; AAT_10_ (**i**–**l**): hybridisation pattern of repetitive microsatellite motif (AAT)_10_, showing abundant accumulation on golden perch Y chromosome; CGG_10_ (**m**–**p**): hybridisation signals from repetitive microsatellite motif (CGG)_10_, showing high accumulation on X chromosome of Murray cod compared with that of the Y chromosome. DNA methylation (**q**–**t**): immunofluorescent detection of DNA methylation, showing hypermethylated X chromosome in Murray cod compared with that of the Y chromosome. Scale bar represent 5 µm.

Intraspecies CGH was performed in three females and three males of each species (Table 1). A minimum of 20 cells were examined from each individual (Table 1). A distinct hybridisation signal for fluorescently labelled male genomic DNA was observed in one of the acrocentric pairs from all golden perch males, but not on chromosomes from any females (Figure 4a,b). This male-specific chromosome is therefore a Y chromosome, identifying males as the heterogametic sex in golden perch. For more accuracy, and to control for any sensitivity differences of the different fluorochromes to different filter sets, male and female genomic DNA was reciprocally labelled (reciprocal CGH) and hybridised onto male and female metaphase spreads. A similar pattern of signal was detected, supporting the presence of the Y chromosome. To conclusively identify Y chromosomes in Murray cod, CGH was also performed on samples from both sexes. In addition to dispersed hybridisation signals on all chromosomes, a comparatively strong hybridisation signal for fluorescently labelled male genomic DNA was observed on the short (p) arm of the smaller homologue of the largest chromosome pair in all three males (Figure 4c,d). This hybridisation signal was consistent in the reciprocally labelled experiments, indicating a male heterogametic sex chromosome system (XX/XY) in Murray cod.

### 2.3. FISH Mapping of Telomeric Probe and Microsatellite Motifs

In both species, mapping of telomeric sequences (TTAGGG)_7_ identified terminal hybridisation signals in all chromosomes, with no evidence of interstitial telomeric sequences (ITSs) (Figure 4e–h). In addition, AT-rich (AAT)_10_ and GC-rich (CGG)_10_ microsatellite motifs were hybridised across the genome of both species, predominantly near the centromeric region. A strong banding pattern for (AAT)_10_ was observed on the Y chromosome of golden perch (Figure 4i), with a dominant hybridisation signal evident on both sex chromosomes (X and Y) of Murray cod (Figure 4k,l). For GC-rich (CGG)_10_ microsatellite motif, a strong banding pattern was observed on the X chromosome of Murray cod (Figure 4o,p) and on both X and Y chromosomes in golden perch (Figure 4m,n).

### 2.4. Immunofluorescence Detection of DNA Methylation

DNA methylation is one of the active epigenetic marks that actively regulates gene expression, and is responsible for X chromosome inactivation in mammals. In this study, we performed a DNA methylation experiment using anti-5-methylcytosine to characterize global methylation patterns in golden perch and Murray cod. For both species, genome-wide signals were detected, predominantly in the telomeric regions. A comparatively stronger but equal strength hybridisation signal was detected on both X and Y chromosomes in golden perch, however, in Murray cod, the X chromosome was hypermethylated compared with that of the Y chromosome (Figure 4q–t).

### 2.5. Cross-Species CGH

Cross-species CGH is an effective tool for understanding genomic and sex chromosome homologies in allopolyploids [26,27,28]. The technique has also been used in comparative genome analysis to visualize the chromosomal distribution of conserved DNA sequences between two species. We performed hybridisation of the genomic DNA probe with differential fluorescent labelling on metaphase chromosome of both species. The strongest hybridisation signal was detected near the centromeric region of the chromosomes, with no distinguishable differences in sex chromosomes (Figure S1).

## 3. Discussion

**Figure 5:**
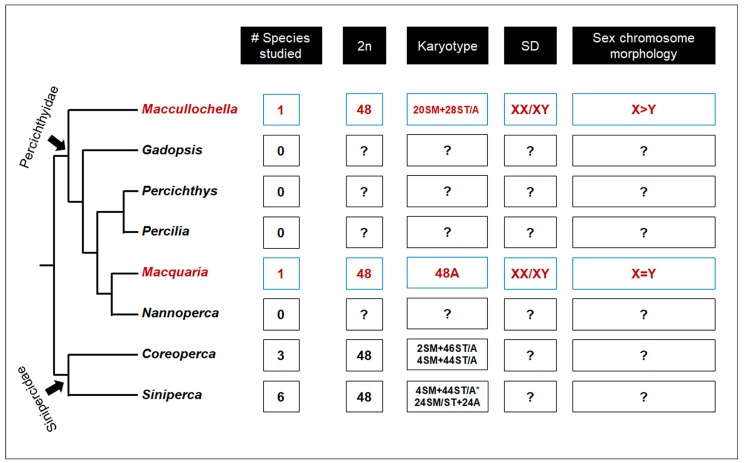
Truncated phylogenetic tree of the family Percichthyidae and sister clade from family Sinipercidae. Three monotypic genus, *Bostockia*, *Guyu* and *Nannatherina* were excluded due to unresolved phylogenetic position. Truncated phylogeny based on 10 nuclear genes was adopted from Near, et al. [29]. Red colour represents information derived from the present study. Phylogeny is not to scale. Asterisk (*) in *Siniperca* genus indicates most frequently observed karyotype [24].

This study is the first karyotypic report of the two Australian native freshwater fish species from the family Percichthyidae, revealing conserved chromosome numbers, 2*n* = 48, in both species. However, we detected a substantial variation in chromosome morphologies (e.g., number of submetacentric and subtelocentric/acrocentric chromosomes) between the studied species; suggesting major chromosome rearrangements. We could not, however, conclusively predict the directionality of the evolution of such chromosome rearrangements because of a lack of karyotypic data from outgroup taxa (Figure 5).

The analysis of patterns of heterochromatin distribution is a useful tool for investigating karyotype diversification in vertebrate species. Within vertebrates, fish tend to lose substantial heterochromatin in their genomes [30]. Our C-banding identified significant C-bands near the centromeric region of the karyotype in both species. There were obvious differences in C-banding patterns in the Murray cod karyotype compared with that of the golden perch. Furthermore, within the Murray cod karyotype, we detected a strong banding pattern in a number of subtelocentric/acrocentric chromosomes. Such diversification in heterochromatin block can be explained by the abundance and variable function of transposable elements.

We used molecular cytogenetic techniques combined with differential banding to search for cryptic sex chromosomes in golden perch and Murray cod. C-banding is effective in revealing sex chromosomes in vertebrates because of the possible large accumulation of heterochromatin in the sex chromosomes [31,32,33]. Along with C-banding we used comparative genome hybridisation (CGH), which has been found to be effective in identifying sex chromosomes in divergent taxa including fish and reptiles [33,34,35]. We identified a male-specific submetacentric chromosome pair as the sex chromosome of Murray cod and a male-specific acrocentric pair of golden perch chromosomes. This suggests that both species have a male heterogametic sex chromosome system (XX/XY). Sex chromosomes of Murray cod can be characterised as having tiny size variation between the X and Y chromosomes (shorter Y), along with an accumulation of heterochromatin on the short arm of both X and Y chromosomes. Sex chromosomes of golden perch are characterised as a homomorphic acrocentric pair, with minor accumulation of heterochromatin at the pericentromeric region.

The mechanism behind sex chromosome evolution remains yet a mystery. Sex chromosomes are thought to have evolved from an autosomal pair through acquisition of a sexually advantageous mutation, which has subsequently been selected in favour of either maleness or femaleness via suppression of recombination, leading to the degeneration of Y or W chromosomes [36,37,38,39]. Accumulation and amplification of repetitive sequences on the sex chromosomes, particularly microsatellites, is thought to be one of the drivers that trigger this process because of their ability to expand rapidly in comparison with other classes of repetitive DNA [40,41,42,43,44]. Compared with birds and mammals, fishes often possess homomorphic sex chromosomes, which have been hypothesized to display high rate of turnover events, where previously established sex chromosomes are being replaced with new sex chromosomes including novel sex-determining genes [45,46]. Our study provides evidence of cryptic sex chromosome turnover between these two species based on the morphological variation, variability in accumulation of heterochromatin and amplification of microsatellite associated repetitive sequences on the X and Y chromosomes. However, many species within the nine genera are yet to be investigated to provide more evidence for the mechanisms and directionality of sex chromosome turnover within the family Percichthyidae.

We observed random accumulation of AT- and GC-rich tri-nucleotide microsatellite motifs ((AAT)_10_ and (CGG)_10_) across the genomes of both species, providing insights into the abundance of repetitive sequences including transposable elements. A distinct variation in the amplification of tri-nucleotide motifs (AAT)_10_ and (CGG)_10_ could indicate karyotype rearrangement between both species. In general, tandem repeats (microsatellite) of small stretches of DNA motifs are widespread in the genomes of eukaryotes. Furthermore, several di-, tri- and tetra-nucleotide microsatellite repeats have been identified on the sex chromosomes of fishes [43,47,48] and amniotes [3,49,50]. The widespread association of microsatellite proliferation within sex chromosomes suggests that such proliferation is one of the likely mechanisms that drive sex chromosome differentiation via suppression of recombination and possible evolution of differentiated sex chromosomes [41]. Future studies on the accumulation of such repeats in other percichthyids may provide a better understanding of karyotype evolution in this family. In addition, we also detected hybridisation signals from conserved vertebrate telomeric repeat (TTAGGG)_7_ in terminal locations of all chromosomes in both species, as expected. However, it is intriguing that we did not detect any interstitial telomeric (ITS) signals on any chromosomes in either of the species given the stark morphological differences in chromosome morphologies between these two species. Accumulation of telomeric repeats in nontelomeric regions signifies evidence of ancestral chromosome rearrangement events. Further studies including other species from this family will be required to provide conclusive evidence on the evolution of morphologically divergent yet conserved karyotypes in this group.

Comparative analysis using cross-species CGH revealed clear banding in the centromeric position of chromosomes from both species (Figure S1). This could be because of a shared accumulation of repetitive sequences in both species. Apart from the centromeric sequences, there was no evidence for common shared genomic regions (entire chromosome or any arm of the chromosome), indicating a variant evolutionary trajectory for these species, even though they belong to the same phylogenetic lineage.

## 4. Materials and Methods

### 4.1. Specimen Collection and Mitotic Chromosome Preparation

Eight Murray cod (five males and three females) were sourced from a hatchery (Murray Darling Fisheries, Narrandera, Australia), and 24 golden perch (12 male and 12 female) were sourced from the Lachlan River (Murray Darling Basin, Australia). For Murray cod, mitotic chromosomes were obtained from kidney and liver cells after mitotic stimulation with yeast, using the protocol described in Bertollo et al. [51]. Briefly, yeast was conjugated with dextrose, diluted in distilled water (1.25 g yeast/0.75 g dextrose/2.5 mL distilled water), homogenized using a sterile spoon spatula, and incubated at 37 °C for 30 min to activate the yeast cells. The solution was homogenized again, and 200 μL were injected into muscle on the caudal peduncle. The specimen was kept in a clean glass tank under optimal condition for 24 h before sacrifice. The fragments of kidney and liver tissues were first dissociated in RPMI 1640 cell culture medium (Life Technologies, Inc., Scoresby, Victoria, Australia), then 160 μL of colchicine (0.0125%) was added and the culture was incubated for 30 min at room temperature. Finally, the cells were subjected to hypotonic treatment in 0.075 M KCl for 30 min at 37 °C and then fixed in Carnoy’s solution (methanol:acetic acid 3:1). Mitotic chromosomes of golden perch were prepared from regenerated fin clips following the protocol described by Völker and Ráb [52]. The hind most part of the caudal fin was cut using sterile dissecting scissors, after which the fish was maintained under optimal condition for three to four days. The regenerated fin material was cut off and treated with Ringer solution (1.281 M NaCl, 0.025 M KCl, 0.018 M CaCl_2_, 0.002 M NaHCO_3_) containing 0.05% colchicine. After two hours of incubation, cells were fixed in Carnoy’s solution.

Cell suspensions were dropped onto glass slides and air-dried. For DAPI (40-6-diamidino-2-phenylindole) staining, slides were mounted with antifade medium Vectashield (Vector Laboratories, Burlingame, CA, USA) containing 1.5 mg/mL DAPI.

### 4.2. C-Banding

Detection of centromeric heterochromatin (C-banding) was performed following the protocol described by Salvadori, et al. [53]. Briefly, 20–25 µL of cell suspension was dropped on slides and air dried and aged on a 60 °C hot plate for 60 min. Slides were then treated with 0.2 M HCl at room temperature for 20 min and with 5% Ba(OH)_2_ at 45 °C for 3 min. Then, the slides were incubated in 2× saline sodium citrate (SSC) at 65 °C for 60 min. Finally, the chromosomes were stained with antifade medium Vectashield containing 1.5 mg/mL DAPI.

### 4.3. Genomic DNA Extraction

Total genomic DNA was extracted from whole blood and fin clips, following the protocol of Ezaz, et al. [54]. Briefly, 25 mg of tissue sample was incubated overnight at 56 °C with lysis buffer (100 mM Tris-HCL, pH 8.0; 10 mM EDTA; 250 mM NaCL), 10% (*w*/*v*) SDS (sodium dodecyl sulphate) and proteinase K. Lysed tissue was incubated at 37 °C for 60 min with 10 µL RNAse A (20 mg/mL), followed by a room temperature incubation for 20 min with 500 µL buffered phenol. After the incubation, the mixture was centrifuged at 16,000× *g* for 12 min, and the supernatant was transferred to a new sterile tube. This step was performed twice, and 500 µL of 24:1 (*v/v*) chloroform:isoamyl alcohol was added to the solution. The solution was incubated at room temperature for 20 min. After the incubation, the mixture was centrifuged twice again, as previously described, and the supernatant was subsequently transferred to a new sterile tube. Then, 0.6–0.8 volume of chilled isopropanol was added to the solution and shaken vigorously for 60 s. The precipitated DNA was washed with 70% ethanol and dried. Finally, the pallet was resuspended in elution buffer (10 mM Tris-HCl, pH 8.0; 0.1 mM EDTA) for 30 min at 50 °C and kept at 4 °C.

### 4.4. Comparative Genomic Hybridisation (CGH) and Cross-Species CGH

We followed the procedure of comparative genomic hybridisation and cross-species CGH described by Ezaz, et al. [33] and Symonová, et al. [55]. Briefly, female and male total genomic DNA was labelled by Nick translation (NT), incorporating Spectrum-Green and Spectrum-Red dUTP, respectively, NT kit (Abbott Molecular, Macquarie Park, Australia). Nick translation reaction was incubated at 15 °C for 2 h. After incubation, NT labelled DNA was checked using 1% agarose gel electrophoresis for size fractionation to 200–500bp. Labelled DNA was co-precipitated by overnight incubation at −20 °C with 5–10 µg of salmon sperm DNA, 20 µg glycogen and 3 volumes of chilled 100% ethanol. Precipitation reaction was centrifuged at maximum speed for 30 min, and the supernatant was discarded and air-dried. Depending on the size of pellet, the co-precipitated probe DNA was resuspended in 30–40 µL hybridisation buffer (50% formamide, 10% dextran sulfate, 2× SSC, 40 mmol/L sodium phosphate pH 7.0 and 1× Denhardt’s solution). The hybridisation mixture was denatured at 70 °C for 10 min, then chilled on ice for 2 min. Subsequently, 18 µL of probe mixture was placed as a single drop on a slide and hybridised at 37°C for 3 days in a humid chamber. Slides were washed once at 60 °C in 0.4× SSC, 0.3% Igepal for 2 min, followed by another wash at room temperature in 2× SSC, 0.1% Igepal. Slides were then air-dried and mounted with antifade medium Vectashield containing 1.5 mg/mL DAPI. Images were captured using a Zeiss Axioplan epifluorescence microscope equipped with a CCD (charge-coupled device) camera (RT-Spot), (Zeiss, Oberkochen, Germany) using filters 02, 10 and 15 from the Zeiss fluorescence filter set or the Pinkel filter set (Chroma technologies, filter set 8300, Bellows Falls, VT, USA). ISIS scientific imaging software (Metasystems, Altlussheim, Germany) was used for image capture and analysis, including karyotyping. For FISH and CGH image analysis, multiple functions of ISIS scientific imaging software were used, such as signal normalizing and background correction. Processed images were pseudocoloured and superimposed, using ISIS scientific imaging software.

For cross-species CGH, golden perch gDNA was labelled with Spectrum-Red dUTP while Murray cod gDNA with Spectrum-Green dUTP using NT, as described above. DNA was coprecipitated with 5–10 µg of salmon sperm DNA (as competitor) and 20 µg glycogen (as carrier). Finally, slides with metaphase spread and labelled genomic DNA were hybridised at 37 °C inside a humid chamber for 3 days. Washing of slides and mounting with antifade medium was carried out in the same way as for the CGH experiments.

### 4.5. Probes for FISH (Fluorescence In Situ Hybridisation) Experiments

The 5’-Cy3-labeled telomeric probe (TTAGGG)_7_ and oligonucleotides of two microsatellite motifs, (AAT)_10_ and (CGG)_10_ were purchased from GeneWorks (Thebarton, South Australia, Australia) to be used as probes. Repetitive DNA mapping FISH was performed under high stringency conditions on metaphase chromosomes, as described by Bonillo, et al. [56]. After denaturation in 70% formamide/2× SSC at 70 °C, chromosomes were dehydrated in a series of ethanol washes. Approximately 500 ng probe for each slide was resuspended in 15 µL hybridisation buffer (50% formamide, 10% dextran sulfate, 2× SSC, 40 mmol/l sodium phosphate pH 7.0 and 1× Denhardt’s solution). The probe mixture was denatured at 70 °C for 10 min, then chilled on ice for 2 min. Denatured probe was subjected to metaphase chromosomes and hybridised at 37 °C for 48 h in a humid chamber. Similar to CGH, slides were washed once at 60 °C in 0.4× SSC, 0.3% Igepal for 2 min, followed by another wash at room temperature in 2×SSC, 0.1% Igepal. Slides were then air-dried and mounted with antifade medium Vectashield containing 1.5 mg/mL DAPI.

### 4.6. Immunofluorescence Detection of DNA Methylation

Immunostaining for DNA methylation was performed following published protocols [57,58], with a slight modification. Briefly, after dehydrating through an ethanol series (70, 90 and 100% *v/v*) metaphase chromosomes were denatured in 70% formamide (*v/v*) in phosphate-buffered saline (PBS: 137 mM NaCl, 2.7 mM KCl, 10 mM Na_2_HPO_4_, 2 mM 2.4 KH_2_PO_4_,) at 70 °C for 2 min. Slides were then quenched in ice cold 70% (*v/v*) ethanol for 5 min, then dehydrated in 90% (*v/v*) and 100% (*v/v*) ethanol for 3 min each before being air-dried. After incubating with PBS-T (PBS with 0.03 % (*v/v*) Tween 20) for 3 min, slides were blocked by incubating in PBS-T with 1% (*w/v*) bovine serum albumin (BSA) for 20 min at room temperature. Metaphase chromosomes were treated with anti-5-methylcytosine primary antibody (1:200 in PBS-T, Clone 10G4, Zymo Research, Irvine, CA, USA) and incubated in a humid chamber at 37 °C for 60 min. Slides were washed twice for 5 min in PBS-T and subjected to 200 μL of the secondary probe (AffiniPure Donkey Anti-Mouse IgG in PBS-T; Jackson ImmunoResearch Laboratories, West Grove, Pa, USA), diluted 1:500 in PBS-T. After 60 min second incubation in a humid chamber at 37 °C, slides were washed twice for 5 min each in PBS-T. Metaphase chromosomes were incubated in 4% (*w/v*) paraformaldehyde at room temperature for 15 min and washed three times (3 min each) in PBS-T. Air-dried metaphase chromosomes were mounted with DAPI (4’,6-diamidino-2-phenylindole) in Vectashield (Vector Laboratories Inc., Burlingame, CA, USA).

## 5. Conclusions

The present study demonstrated the successful use of both conventional (mitotic chromosome preparation, C-banding) and advanced (CGH, FISH, cross-species CGH) cytogenetic techniques for two Australian freshwater fishes to characterise karyotypes. This information provides critical baseline data for future cytogenetic analysis of fishes, especially in the family Percichthyidae. The identification of sex chromosomes is a first step in understanding the evolution of sex determination mechanisms in these fishes, which occupy highly hydrologically variable habitats. In addition, information on sex chromosomes provides the basis for developing nondestructive methods for sex identification of fish in hatcheries, allowing management of sex ratios and identifying the probability of sex reversal.

## Figures and Tables

**Table 1:** Number of individuals and cells examined for the two fish species. The number of cells in the column for each group refers to the least number of mitotic chromosomes examined. CGH = Comparative Genomic Hybridisation; FISH = Florescence in situ hybridisation.

	Golden Perch				Murray Cod			
	Female		Male		Female		Male	
	Number of individuals	Number of cells/each	Number of individuals	Number of cells/each	Number of individuals	Number of cells/each	Number of individuals	Number of cells/each
Mitosis	12	60	12	60	3	60	5	60
Karyotyping	5	20	5	20	3	20	5	20
CGH	3	20	3	20	3	20	3	20
C-banding	3	60	5	60	3	60	5	60
FISH- telomeric probe	3	20	3	20	3	20	3	20
FISH-repetitive sequences	3	20	3	20	3	20	3	20
DNA methylation	3	20	3	20	3	20	3	20
Cross-species CGH	-	-	1	20	-	-	1	20

## Supplementary Materials

Supplementary materials can be found at https://www.mdpi.com/1422-0067/20/17/4244/s1.
