# Supplementary material for: Karyotypes and Sex Chromosomes in Two Australian Native Freshwater Fishes, Golden Perch (Macquaria ambigua) and Murray Cod (Maccullochella peelii) (Percichthyidae)
Source: Int J Mol Sci. 2019 Aug 30;20(17):4244. doi: 10.3390/ijms20174244 (PMC6747191; doi:10.3390/ijms20174244)
Supplement: Supplementary file 1 [file ijms-20-04244-s001.pdf]

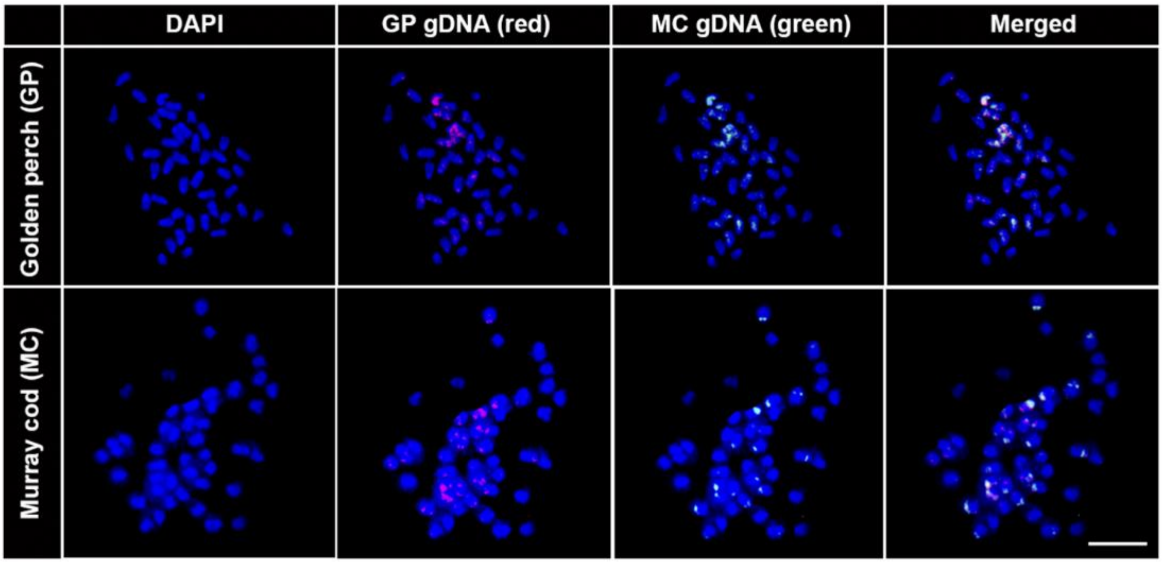

Figure S1. Cross-species CGH of golden perch and Murray cod. Red signals represent hybridisation of golden perch genomic DNA and green signals represents hybridisation pattern of Murray cod in metaphase chromosome of both species. Scale bar 5 μm
